# Double-Blind Randomized Controlled Trial Comparing Platelet-Rich Plasma With Intra-Articular Corticosteroid Injections in Patients With Bilateral Knee Osteoarthritis

**DOI:** 10.7759/cureus.29744

**Published:** 2022-09-29

**Authors:** Jacques Pretorius, Nouman Nemat, Almutaz Alsayed, Ahmed Mustafa, Yasir Hammad, Tony Shaju, Sayed Nadeem

**Affiliations:** 1 Trauma and Orthopaedics, Letterkenny University Hospital, Letterkenny, IRL; 2 Trauma and Orthopaedics, University Hospital Waterford, Waterford, IRL

**Keywords:** knee osteoarthritis, platelet-rich plasma (prp), randomized controlled trial, bilateral knee involvement, corticosteroid injection

## Abstract

Introduction

Platelet-rich plasma (PRP) intra-articular injections have gained popularity and are suggested to be more effective and longer lasting than corticosteroid or visco-supplementation therapy. There are few studies comparing PRP with corticosteroid injections and none comparing PRP in patients with bilateral knee osteoarthritis with the patient acting as their own control.

Methods

We performed a double-blind randomized controlled trial including 29 patients (58 knees) with radiologically confirmed mild-to-moderate bilateral knee osteoarthritis. They were randomized to receive an intra-articular PRP injection into one knee and a methylprednisolone injection with a local anesthetic into the contralateral knee. The primary outcome was measured using the Western Ontario and McMaster Universities Arthritis Index (WOMAC) before the treatment and at six weeks, three months, and six months. Secondary outcome was measured pain with the visual numerical pain rating scale (VNS).

Results

Corticosteroids and PRP were both effective in improving pain, stiffness, and function at all time points, with maximal improvements at six weeks and three months. PRP scored slightly better than steroid injections at six months; nevertheless, there was no statistically significant difference between corticosteroids and PRP injections (F_2,139_=0.173, p=0.84). The secondary outcome also delivered the same result with improvement at all time points but no statistically significant difference (F_2,139_=0.168, p=0.85).

Conclusions

Both corticosteroids and PRP interventions are effective in improving pain, stiffness, and function in patients with bilateral knee osteoarthritis up to six months with no statistically significant difference between the two.

## Introduction

Osteoarthritis (OA) is the most common cause of arthritis, disability, and knee pain in elderly patients [1]. Furthermore, knee OA is the most common OA involving the lower limb and constitutes 23% of all arthritis cases [2]. Up to 10% of all people above the age of 55 years suffer from OA of the knees, with a large number of these patients suffering with significant mobility restriction [3]. The pathological basis of OA is an imbalance of the homeostasis between cartilaginous matrix synthesis and degradation [4]. The main cause of the patients’ symptoms is the progression of articular chondral lesions; these symptoms include stiffness, edema, decreased range of motion, and nocturnal pain [5]. This generally becomes significant enough that it leads to deterioration in the patient’s quality of life and emotional well-being.

Currently, no disease-modifying agents are available for the treatment of OA; therefore, the only definitive treatment available is surgical management, which entails a total knee arthroplasty [6]. Prior to surgical intervention, physicians would attempt to control symptoms with conservative measures, which include patient education, exercise, weight loss, walking aids, bracing, acupuncture, and electromagnetic therapy [7]. In the early stages of OA, a combination of non-pharmacological and oral medications can provide sufficient relief of symptoms, but pharmacological therapy alone is generally insufficient for the desired symptom control [3].

Intra-articular injections would usually be reserved for patients who do not have adequate relief of symptoms on oral treatment regimens [8]. Hyaluronic acid injections are commonly used, but they are generally discouraged because of the high risk of adverse effects and no significant benefit over steroid injections [9]. A recent meta-analysis on intra-articular platelet-rich plasma (PRP) and steroid injections suggested that there is a favorable outcome of PRP when compared to corticosteroid injections with maximal difference at six months’ follow-up [10]. Despite these promising results, PRP has still not gained acceptance and the confidence of orthopedic surgeons as a valid treatment option. This might also be due to our lack of knowledge regarding PRP segregation techniques as well the lack of standardization of study protocols and outcome analysis [11]. There are numerous studies available comparing PRP and hyaluronic acid injections, whereas the number of studies comparing PRP and corticosteroid injections is limited in comparison. There are no studies available comparing the effects of PRP with steroid injections in bilateral knee OA in the same patient. In this study, we assessed whether PRP intra-articular injections are more effective than steroid injections in treating patients with mild-to-moderate knee OA in relation to pain, stiffness, and function.

## Materials and methods

This is a single-center, prospective, double-blind randomized controlled trial performed in the Orthopedic Department at Letterkenny University Hospital between August 2021 and April 2022. This study included 31 patients and therefore 62 knees (14 males and 17 females; mean age: 64.2±9.9 years) diagnosed with primary bilateral knee OA and classified according to the Kellgren-Lawrence classification scale (Table 1).

**Table 1:** Proportion of patients with different grading by Kellgren-Lawrence scale assigned to each therapy

Kellgren and Lawrence grade	Platelet-rich plasma (%)	Corticosteroid (%)
II	8 (28)	10 (34)
III	21 (72)	19 (66)

**Figure 1:**
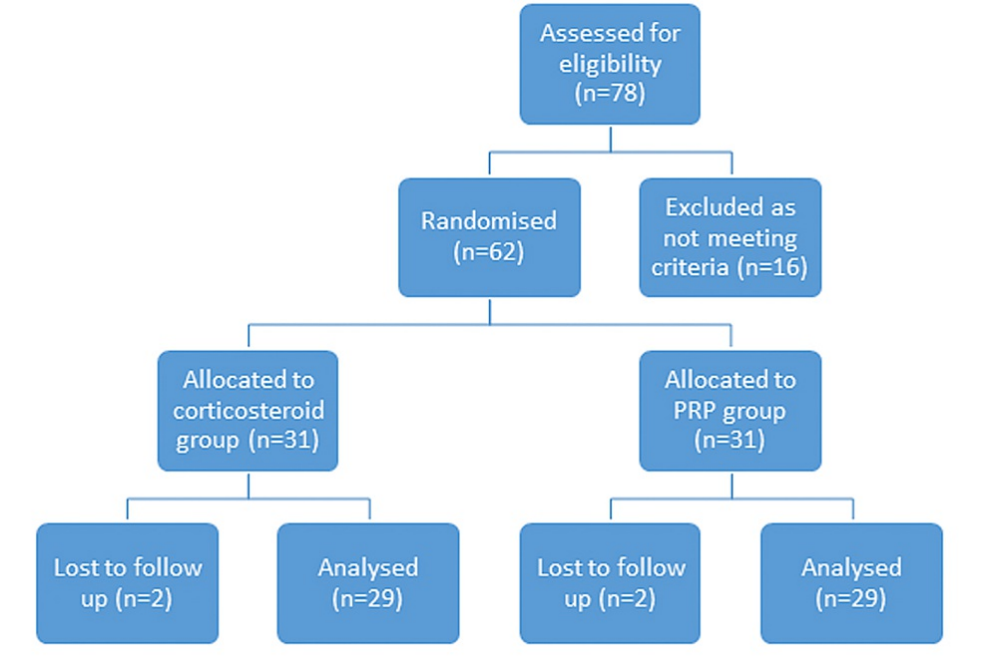
Flowchart of enrolment n, number of patients

The study had two patients who were lost to follow-up, and the study was therefore completed with 29 patients/58 knees (12 males and 17 females; mean age: 63.8 ± 9.7 years; BMI: 32.7 ± 4.9). Of these 29 patients, each patient received a PRP injection into one knee and a corticosteroid injection into the contralateral knee. The knees receiving the PRP injection comprised the intervention group and the knees receiving the corticosteroid injection comprised the control group (Figure 1).

The sample size was determined using the calculation performed by Freire et al., where the number of components in each sample was stipulated to achieve a 95% confidence interval, 80% power, and a 20% difference between groups; to achieve this, the intervention and control group should each consist of 25 knees with a total of 50 knees [12]. We therefore decided to include >60 knees to accommodate any loss to follow-up and to ensure that our sample size would still be sufficient.

The inclusion criteria were patients with bilateral knee grade II/III OA according to the Kellgren-Lawrence scale grade and age ranging from 40 to 80 years. The exclusion criteria include other rheumatological inflammatory conditions, previous intra-articular injection in the last six months, cancer, acute infection, pregnancy and breastfeeding, BMI > 40, blood disorders, uncontrolled diabetes mellitus, hemoglobinopathies, treatment of coagulation disorders, previous knee surgeries, and liver disease.

The study protocol was approved by the Ethics Committee of Letterkenny University hospital (Approved date: October 23, 2020). A written informed consent was obtained from each patient prior to participation in the study.

The interventions were randomized using simple randomization. This was performed with a coin toss to determine the intervention group (PRP injection). If it landed on heads, then PRP would be injected into the right knee and tails the left knee. The contralateral knee would then be injected with a corticosteroid.

During the PRP preparation, 15 mL of blood was taken from the antecubital fossa of each patient under sterile conditions with an Arthrex ACP® double syringe system (Arthrex, Munich, Germany). This was then inserted into a Rotofix 32A centrifuge with a counterweight and centrifuged for 5 minutes at 6,000 rpm. Once removed, the autologous PRP was then drawn up from the inner syringe, which would usually be around 5 mL for each patient, which would then be injected into the knee joint.

The corticosteroid injection used was 80 mg (2 mL) of methylprednisolone acetate injectable suspension (Depo-Medrol) with 40 mg of levobupivacaine (8 mL of 0.5% chirocaine). Patients were then positioned supine with both knees flexed up to 70° with a sheet divider blocking the patients’ view of the interventions given (blinding the patients). After randomization, the PRP and corticosteroid were injected in the appropriate knees with an inferolateral approach.

The intensity of pain and knee function were evaluated based on the Western Ontario and McMaster Universities Arthritis Index (WOMAC) and the visual numerical pain rating scale (VNS) before the onset of treatment and at six weeks, three months, and six months after initial treatment. In the VNS, a score of 0 represents no pain at all and a score of 10 indicates highest intensity of pain ever experienced. The WOMAC score consists of three sections, which include pain, stiffness, and a function scale. The two experienced physicians who recorded the initial as well as follow-up scoring were blinded to the interventions given to each knee. The treatments were labelled as treatment A (PRP) and treatment B (corticosteroid). The statistician was also blinded to the treatment as she was only given the data to analyze for treatments A and B.

To perform the statistical analysis, a linear mixed effects model with random patient effects was used. This method was employed as patients were given both corticosteroids and PRP into contralateral knees, and there were repeated measurements of the outcome over time. Adjustments were made for differences in the baseline value, of the outcome score, by including it as a covariate in the model. An interaction term between treatment and time was included (modelled as a categorical variable) to assess evidence of a difference in treatment effects at different time points. Following this, 95% confidence intervals were measured by bootstrapping. F statistics were reported from the ANOVA tables for the linear mixed effects models. The statistical analyses were conducted using R Version 4.1.0 (R Foundation for Statistical Computing, Vienna, Austria) and the lme4 package [13].

## Results

Corticosteroids and PRP were both effective in improving pain, stiffness, and function at all of the different timeframes, with maximal improvements at six weeks and three months (Table 2).

**Table 2:** Time-dependent changes in the WOMAC scores of PRP and corticosteroids interventions PRP, platelet-rich plasma; SD, standard deviation; WOMAC, Western Ontario and McMaster Universities Arthritis Index

WOMAC score and time	Baseline (mean ± SD)	6 weeks (mean ± SD)	3 months (mean ± SD)	6 months (mean ± SD)
Pain				
PRP	11.75±4.61	9.45±5.79	9.83±5.66	10.9±5.55
Steroid	11.34±4.17	8.83±5.43	9.17±5.83	10.83±5.27
Stiffness				
PRP	5.55±1.79	3.93±2.41	4.24±2.65	5.07±2.16
Steroid	5.41±1.65	3.48±2.31	4.31±2.52	5.14±2.3
Function				
PRP	43.62±12.66	34.03±20.81	34.17±19.21	40.1±19.23
Steroid	42.34±12.92	33.03±19.43	34.66±19.91	41.0±18.72
Total				
PRP	60.93±17.55	47.41±28.37	48.24±26.71	56.03±26.4
Steroid	59.1±17.67	45.24±26.18	48.14±27.83	56.9±25.63

**Figure 2:**
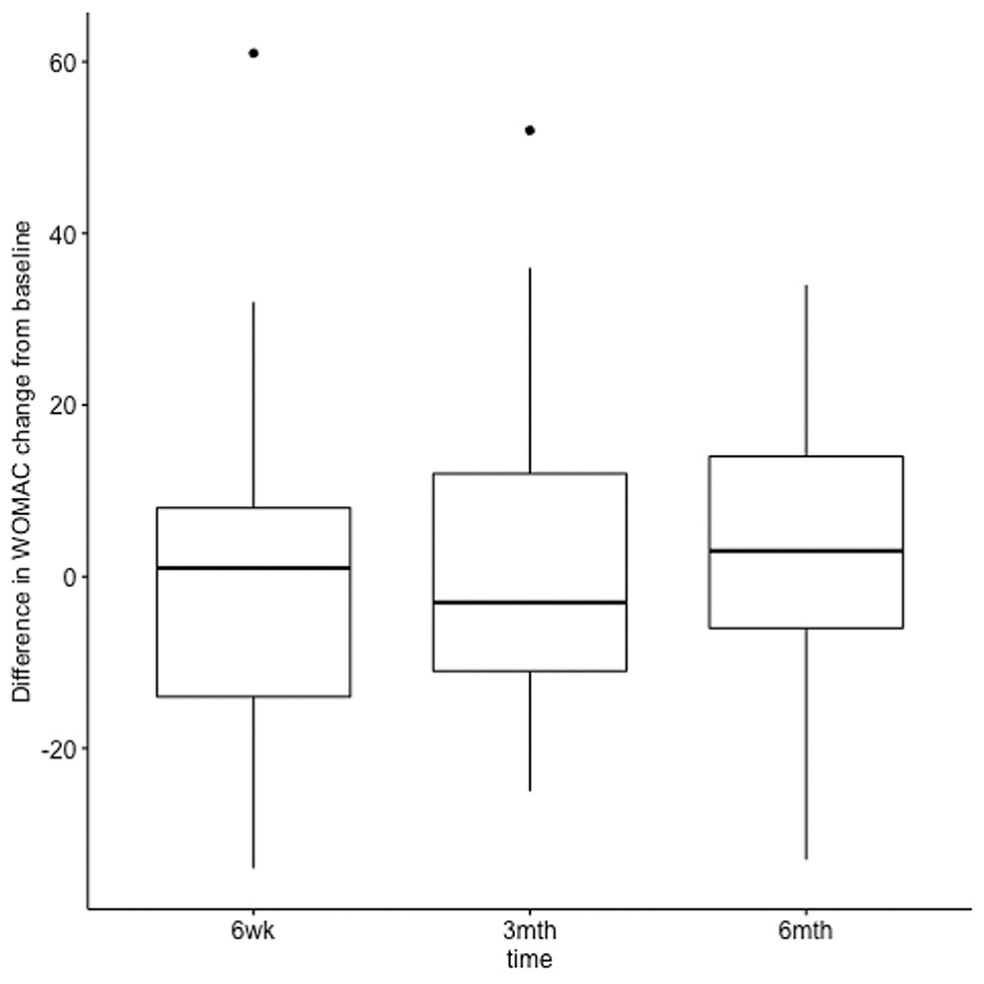
Difference from baseline in WOMAC between platelet-rich plasma versus corticosteroid injections WOMAC, Western Ontario and McMaster Universities Arthritis Index

The data showed no evidence of a difference in treatment (PRP vs corticosteroid) effects at different times for the total WOMAC score (F_2,139_=0.173, p=0.84) (Figure 2).

Looking at the main effects of treatment and time, we found no evidence of a treatment effect (F_1,139_=0.131, p=0.72). As mentioned before, there was a statistically significant improvement in WOMAC score over time for both treatments (F_2,139_=8.88, p=0.0002). PRP scored slightly better than steroid injections at six months, although no statistically significant difference was identified between corticosteroids and PRP intervention at six months’ follow-up. In both interventions, total WOMAC scores worsened from three months (PRP=48.24 and steroids=48.14) to six months (PRP=56.03 and steroids=56.9) but still scored better than baseline (PRP=60.93 and steroids=59.1) (Table 3).

**Table 3:** Primary and secondary outcomes with statistical analysis ns, p > 0.05; VNS, visual numerical pain rating scale; WOMAC, Western Ontario and McMaster Universities Arthritis Index

		PRP (n=29)		Corticosteroid (n=29)			
	Weeks	Mean ± SD	% Improvement	Mean ± SD	% Improvement	CI (95%)	p-value (intergroup)
WOMAC Total							
W1	0	60.93±17.55		59.1±17.67			ns
W2	6	47.41±28.37	22.1%	45.24±26.18	23.5%	-8.15 to 6.67	ns
W3	13	48.24±26.71	20.9%	48.14±27.83	18.4%	-5.63 to 7.79	ns
W4	26	56.03±26.4	8.1%	56.9±25.63	3.9%	-5.64 to 8.83	ns
VNS							
V1	0	6.69±2.01	18.6%	6.69±2.17			ns
V2	6	5.45±2.97	18.6%	4.97±3.13	25.8%	-1.46 to 0.63	ns
V3	13	4.93±2.96	26.4%	4.86±3.2	27.3%	-1.00 to 0.88	ns
V4	26	6.17±2.53	7.8%	5.97±2.66	10.8%	-1.28 to 0.76	ns

**Figure 3:**
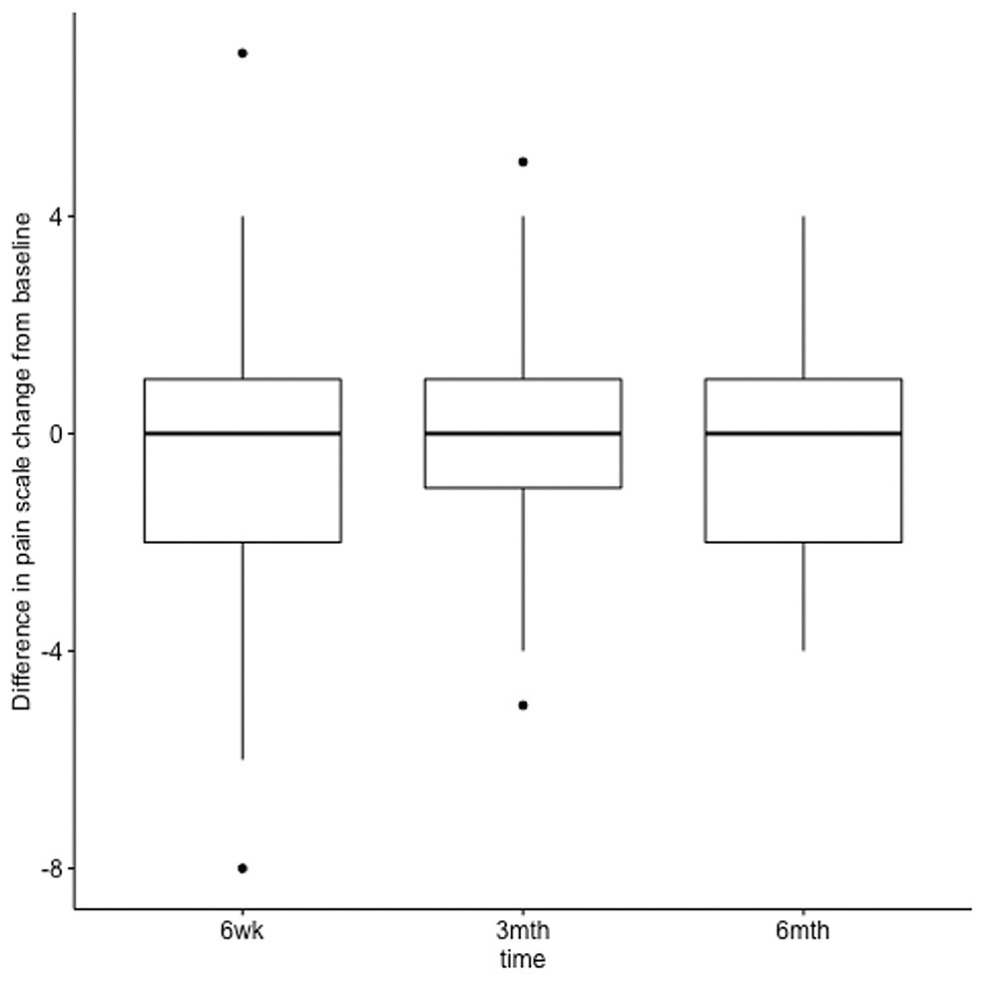
Difference from baseline in VNS between platelet-rich plasma versus corticosteroid injections VNS, visual numerical pain rating scale

Our secondary outcome (VNS) delivered the same result with improvement of the pain score from baseline to six weeks, three months, and six months with the PRP injection (6.69, 5.45, 4.93, and 6.17, respectively) as well as the steroid injection (6.69, 4.97, 4.86, and 5.97) (Table 2), although we found no evidence of a difference in treatment (PRP vs corticosteroid) effects at different times for the pain score (F_2,139_=0.168, p=0.85) (Figure 3). Looking at the main effects of treatment and time, we also found no evidence of a treatment effect between the two interventions (F_1,139_=0.725, p=0.72). As we would expect, we found statistically significant improvement in pain score over time for both treatments (F_2,139_=5.57, p=0.005).

The patient with Kellgren and Lawrence grade II knees had the best response to both corticosteroid and PRP injection. This patient group had the most significant improvement in WOMAC total score from baseline (60.22) to six weeks (30.22), and the improvement lasted up to six months, with the total WOMAC score still below 45.

## Discussion

Knee OA treatment begins with conservative therapies, which involve oral analgesic agents (NSAIDs), physical therapy, weight loss, patient counselling, and exercise. As the disease progresses, other therapeutic methods become important such as intra-articular injections, which include corticosteroids, hyaluronic acid, and PRP [12]. There is still ongoing controversy regarding which injection is the most effective treatment modality.

A recent systematic review performed comparing intra-articular PRP and steroid injections for the management of symptomatic knee OA showed that PRP has an overall greater efficacy compared to corticosteroid injections over 12 months’ follow-up. Although it is important to note that seven of the eight studies assessed in this study followed up patients for only six months. This effect was statistically significant from three months onward and is most pronounced at six months’ follow-up. The benefits were greatest with patients with grade II to III OA. PRP showed greater efficacy in reducing pain scores while also improving stiffness and general physical function [10]. In contrast to this, our study did not show any statistically significant difference between PRP and corticosteroid injections neither at three months nor at six months. Our study also showed notable worsening in pain and functional symptoms at six months for both the PRP and steroid groups. The benefits experienced by the patients were the most pronounced for grade II OA.

Intra-articular corticosteroid injections are widely used to reduce pain and limitation of joint movement in knee OA, particularly in the presence of inflammation and joint effusion [14]. But two systematic reviews on the use of corticosteroid intra-articular injections for knee OA found that the effect of corticosteroid injections was only significant at one week, with little benefit after a period of six weeks [15,16]. The results of our study contradict this with corticosteroids leading to improvement up to six months, although most significant improvement is at six weeks and three months with a worsening in WOMAC and VNS scores at six months.

PRP injection has been shown to be a safe treatment option with no serious complications. Minor side effects have been reported with repeated intra-articular injections, which include symptoms of pain, swelling, and mild effusion that can last a few days [14]. None of the patients receiving PRP injection complained of any side effects after receiving the injections.

It is important to keep in mind that it has been suggested that PRP injections are more effective in patients with mild-to-moderate OA as well as patients of younger age [17]. Kon et al. suggested that it is most effective in patients under the age of 50 [18]. Our study suggests that there is a significant difference in response to intra-articular injections for patients with grade II rather than grade III Kellgren and Lawrence OA. Our patient demographics involved an older patient profile with a mean age of 63.8 years of age. Therefore, it might be better for future studies to compare a younger population group with grade I and grade II OA to be able to better distinguish between the degree of benefit of PRP versus corticosteroids. It has been suggested that PRP will have a better response in the earlier stages of disease progression due to its ability to restore and protect cartilage, which would be more evident in patients with more preexisting joint cartilage present [19].

It has been suggested that multiple PRP injections likely have a better outcome than single PRP injections [20,21]. This could possibly have resulted in a statistically significant difference in outcome measures if multiple PRP injections were compared to a single corticosteroid injection, although it should be noted that multiple injections might lead to an increased risk of local reactions [22].

This study has several limitations. First, a small sample size was included in the study and the study had a short to midterm follow-up period. Another limitation is that injections were not administered under ultrasound guidance, although we used two experienced orthopedic doctors to administer all the injections with a standardized technique. One of this study’s greatest benefits could also be considered as a limitation, that is, using the patients’ own contralateral knees as their own control group. This removed the interpersonal variations between different patients with regard to patient demographics and comorbidities. But there is a possibility that there might be some carryover effect between the two knees as patients will inadvertently be comparing one knee to the other when reporting on pain, stiffness, and function in the WOMAC as well as VNS scoring systems.

## Conclusions

Both PRP and corticosteroid injections are effective in improving pain, stiffness, and function in patients with bilateral knee OA. The greatest effect is in patients with mild OA for both corticosteroid and PRP injections. The degree of pain and functional improvement reduces at six months postinjection. The PRP group scored slightly better at six months’ follow-up, but this was not statistically significant.

This study adds to the continuing controversy regarding which injectable therapy is the most effective in treating patients with mild-to-moderate knee OA. There is still a need for more larger, well-constructed, multi-center randomized controlled trials comparing PRP and corticosteroid injections with long-term follow-up to attempt to provide more clarity on the matter.
